# Supplementary material for: Regulation of 4-HNE via SMARCA4 Is Associated with Worse Clinical Outcomes in Hepatocellular Carcinoma
Source: Biomedicines. 2023 Aug 16;11(8):2278. doi: 10.3390/biomedicines11082278 (PMC10452552; doi:10.3390/biomedicines11082278)
Supplement: Supplementary file 1 [file biomedicines-11-02278-s001.zip › biomedicines-2534738-supplementary.pdf]

Supplementary data

Regulation of 4-HNE via SMARCA4 Is Associated with Worse Clinical Outcomes in Hepatocellular Carcinoma

Shiori Watabe, Yukari Aruga, Ryoko Kato, Genji Kawade, Yuki Kubo, Anna Tatsuzawa, Iichiroh Onishi, Yuko Kinowaki, Sachiko Ishibashi, Masumi Ikeda, Yuki Fukawa, Keiichi Akahoshi, Minoru Tanabe, Morito Kurata, Kenichi Ohashi, Masanobu Kitagawa, and Kouhei Yamamoto

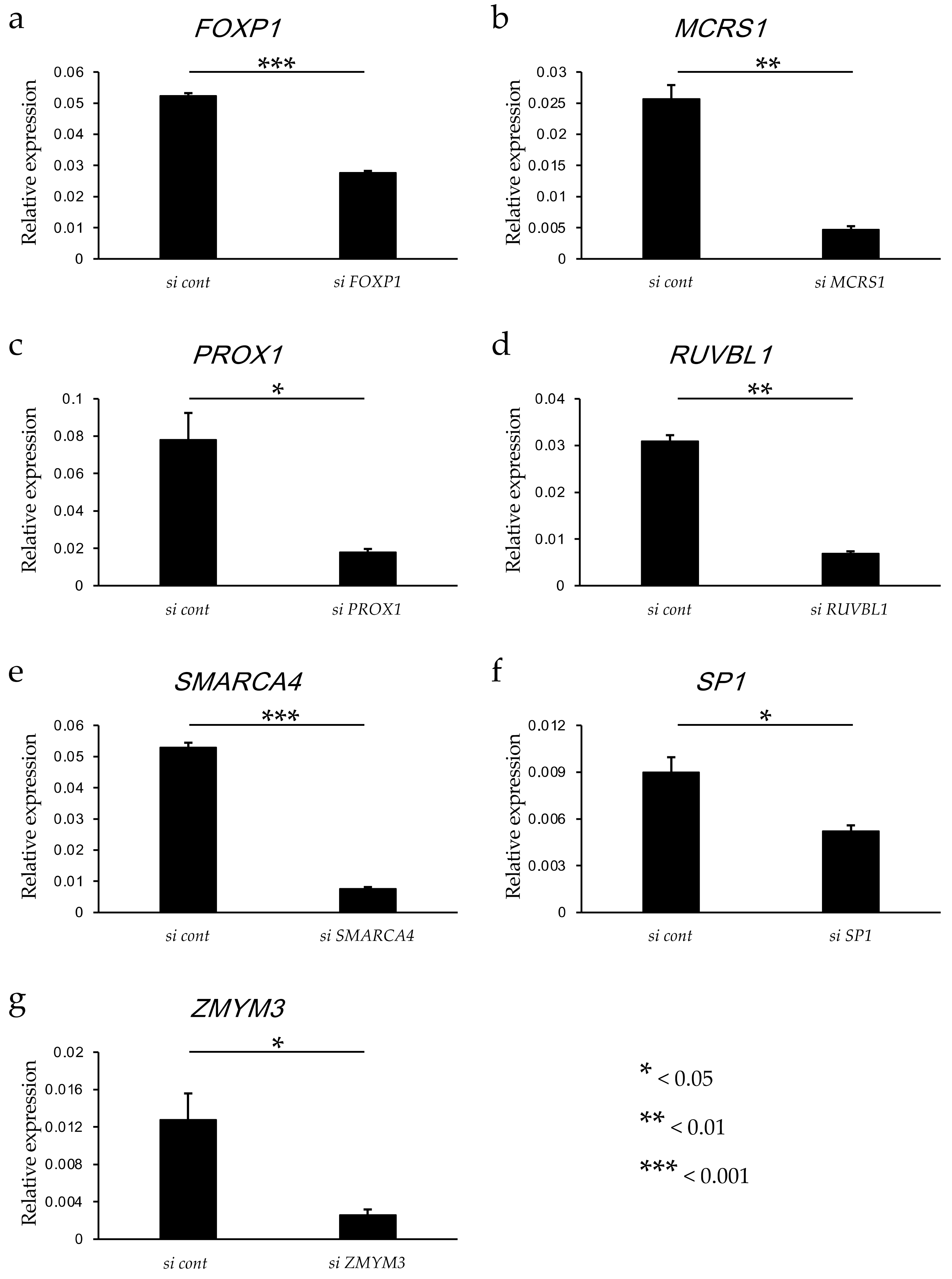

Figure S1: mRNA levels of each gene downregulated using small interfering RNA (siRNA) were measured, and knockdown efficiency was detected by RT-qPCR. All mRNA levels were significantly decreased on knockdown by siRNA transfection. a. *FOXP1* ($p < 0.001$), b. *MCRS1* ($p < 0.01$), c. *PROX1* ($p < 0.05$), d. *RUVBL1* ($p < 0.01$), e. *SMARCA4* ($p < 0.001$), f. *SP1* ($p < 0.05$), and g. *ZMYM3* ($p < 0.05$)

Table S1 Primary antibody and conditions for immunostaining

Antibody					
	**4-HNE**	**GPX4**	**FSP1**	**GCH1**	**SMARCA4**
Source	JaICA	Abcam	ATLAS ANTIBODIES	SIGMA	Proteintech
Clone	HNEJ-2	EPNCIR144	HPA042309	HPA028612	BC150298
Host	Mouse	Rabbit	Rabbit	Rabbit	Rabbit
Clonality	Monoclonal	Monoclonal	Polyclonal	Polyclonal	Polyclonal

IHC condition					
Antigen retreval	MW, 97℃, 20 min	MW, 97℃, 40 min	MW, 97℃, 20 min	MW, 97℃, 20 min	MW, 97℃, 20 min
Buffer	pH6.0 citrate buffer	pH6.0 citrate buffer	pH6.0 citrate buffer	pH9.0 HISTOFINE	pH9.0 HISTOFINE
Concentration	×200	×1000	×500	×500	×400
Method	ABC	Polymer	ABC	ABC	Polymer

Table S2 siRNA sequences for knockdown

	**Sense**	**Antisense**
si FOXP1	5'-GCAAGUUAGUGGAUUAAAATT-3'	5'-UUUUAAUCCACUAACUUGCTG-3'
si MCRS1	5'-GCCUGGAAGAUAUCCCGGATT-3'	5'-UCCGGGAUAUCUUCCAGGCCG-3'
si PROX1	5'-GUUUGAUAUGGAUCGCUUATT-3'	5'-UAAGCGAUCCAUAUCAAACTG-3'
si RUVBL1	5'-GAAGUUUACUCAACUGAGATT-3'	5'-UCUCAGUUGAGUAAACUUCAC-3'
si SMARCA4	5'-GGAAUACCUCAAUAGCAUUTT-3'	5'-AAUGCUAUUGAGGUAUUCCTG-3'
si SP1	5'-GGCAGACCUUUACAACUCATT-3'	5'-UGAGUUGUAAAGGUCUGCCCT-3'
si ZMYM3	5'-CAACAGUUCUCAGACCAATT-3'	5'-UUUGGUCUGAGAACUGUUGAT-3'

Table S3 Primer sequences for quantitative PCR

	Forward	Reverse
FOXP1	5'-GCAGCAGCTCTGGAAAGAAGTG-3'	5'-AGGAGACACATGTCGTGGTCAG-3'
MCRS1	5'-CAAAGGGACCAAGTGCTGAAC-3'	5'-CAGCTCATGTTCCAGGACCTCA-3'
PROX1	5'-ATGAGATGTGCGAGCTAGACCC-3'	5'-CCTTCTCGCTTCGGCTTGTTTT-3'
RUVBL1	5'-CGTGCCCAGACGGAAGGAAT-3'	5'-CAGCAGCTGCACTGAGTACC-3'
SMARCA4	5'-ACCCACCCAACCTCACCAAG-3'	5'-CGAGGGCAGCTGGATGAAGA-3'
SP1	5'-GCACCCAATTCAAGGCCTGC-3'	5'-ATTCCATCACCACCAGCCCC-3'
ZMYM3	5'-GGGGCAAATTCCTGTTCACCTG-3'	5'-ATGGAGGTGGTCTCCTCCTCTT-3'

Table S4 The number of cases of each score in each antibody

	Score 0	Score 1	Score 2
4-HNE	160 (72.4%)	34 (15.4%)	27 (12.2%)
GPX4	34 (15.4%)	110 (50.0%)	77 (34.8%)
FSP1	94 (42.5%)	92 (41.6%)	35 (15.8%)
GCH1	81 (36.7%)	65 (29.4%)	75 (33.9%)

Table S5 Relationship between background liver status and 4-HNE accumulation

		Liver chirrhosis		
		Negative	Positive	P-value
4-HNE	High	40	31	0.7702
	Low	89	61	
		Hepatitis B virus		
		Negative	Positive	P-value
	High	61	10	0.4501
	Low	122	28	
		Hepatitis C virus		
		Negative	Positive	P-value
	High	35	36	0.3153
	Low	85	65	
		Steatosis		
		Negative	Positive	P-value
	High	50	21	0.4067
	Low	89	61	

Table S6 Correlation between 4-HNE accumulation and clinicopathological features

		4-HNE		
**Variable**		**Score 0**	**Score 1,2**	***P*-value**
Age	< 70 y	73	22	0.226
	≥ 70 y	87	39	
Gender	Male	123	41	0.169
	Female	37	20	
ALT	≤ 50 IU/L	110	42	1.000
	> 50 IU/L	50	19	
Cirrhosis	+	65	26	0.879
	−	95	35	
Tumor size	≤ 5 cm	105	41	0.875
	> 5 cm	55	20	
Lesion	Single	106	49	**0.049**
	Multiple	54	12	
Vascular invasion	+	78	16	**0.002**
	−	82	45	
HCV infection	+	69	32	0.230
	−	91	29	
HBV infection	+	31	7	0.231
	−	129	54	
Differentiation	Well	35	25	**0.002**
	Moderate	88	30	
	Poor	37	6	
Stage	Ⅰ， Ⅱ	62	37	**0.004**
	Ⅲ，Ⅳ	98	24	
Ki-67	< 30 %	88	49	**0.001**
	≥ 30 %	72	12	

Table S7 Correlation between GPX4 expression and clinicopathological features

Variable		GPX4 Score 0,1	GPX4 Score 2	*P*-value
Age	< 70 y	63	32	0.777
	≥ 70 y	81	45	
Gender	Male	108	56	0.748
	Female	36	21	
ALT	≤ 50 IU/L	101	51	0.547
	> 50 IU/L	43	26	
Cirrhosis	+	95	35	**0.004**
	−	49	42	
Tumor size	≤ 5 cm	92	54	0.375
	> 5 cm	52	23	
Lesion	Single	104	51	0.359
	Multiple	40	26	
Vascular invasion	+	85	42	0.569
	−	59	35	
HCV infection	+	87	33	**0.016**
	−	57	44	
HBV infection	+	119	64	1.000
	−	25	13	
Differentiation	Well	42	18	0.662
	Moderate	75	43	
	Poor	27	16	
Stage	Ⅰ，Ⅱ	65	34	1.000
	Ⅲ，Ⅳ	79	43	
Ki-67	< 30 %	95	42	0.110
	≥ 30 %	49	35	

Table S8 Correlation between FSP1 expression and clinicopathological features

		FSP1		
**Variable**		**Score 0,1**	**Score 2**	***P*-value**
Age	< 70 y	80	15	1.000
	≥ 70 y	106	20	
Gender	Male	143	21	0.056
	Female	43	14	
ALT	≤ 50 IU/L	128	24	1.000
	> 50 IU/L	58	11	
Cirrhosis	+	111	19	0.578
	−	75	16	
Tumor size	≤ 5 cm	118	28	0.079
	> 5 cm	68	7	
Lesion	Single	129	26	0.688
	Multiple	57	9	
Vascular invasion	+	105	22	0.577
	−	81	13	
HCV infection	+	103	17	0.467
	−	83	18	
HBV infection	+	153	30	0.808
	−	33	5	
Differentiation	Well	48	12	0.184
	Moderate	98	20	
	Poor	40	3	
Stage	Ⅰ，Ⅱ	82	17	0.712
	Ⅲ，Ⅳ	104	18	
Ki-67	< 30 %	114	23	0.706
	≥ 30 %	72	12	

Table S9 Correlation between GCH1 expression and clinicopathological features

Variable		GCH1		
		Score 0,1	Score 2	*P*-value
Age	< 70 y	57	38	0.115
	≥ 70 y	89	37	
Gender	Male	103	61	0.104
	Female	43	14	
ALT	≤ 50 IU/L	105	47	0.170
	> 50 IU/L	41	28	
Cirrhosis	+	92	38	0.085
	−	54	37	
Tumor size	≤ 5 cm	93	53	0.368
	> 5 cm	53	22	
Lesion	Single	93	53	0.368
	Multiple	53	22	
Vascular invasion	+	85	42	0.775
	−	61	33	
HCV infection	+	85	35	0.118
	−	61	40	
HBV infection	+	121	62	1.000
	−	25	13	
Differentiation	Well	39	21	0.981
	Moderate	78	40	
	Poor	29	14	
Stage	Ⅰ， Ⅱ	66	33	0.887
	Ⅲ， Ⅳ	80	42	
Ki-67	< 30 %	96	41	0.143
	≥ 30 %	50	34	
